# Naso-Pharyngeal Syphilis

**Published:** 1901-12

**Authors:** 


### NASO-PHARYNGEAL SYPHILIS.*

BY FISCHENICH.

In two hundred and thirty-five cases of syphilis of the throat, nose and ears treated by the author in the last fifteen years, only thirty-nine times did he find the lesion in the naso-pharynx. In fifteen of these cases the lesion was confined to this region; in the others syphilitic manifestations were elsewhere. In this number the primary lesion did not figure once, though in one case mucous patches were observed on the posterior surface of

*Translated from *La Reone Medicale* by Arthur G. Hobbs, M.D., Atlanta, Ga.

the soft palate in the immediate vicinity of the choanæl. All the other cases presented tertiary manifestations in the naso-pharynx towards the sixteenth year after the infection; the extreme dates were twenty-two years and two and a half years. The anatomical picture of syphilis of the naso-pharynx presents few variations. The point of departure is constituted by a gummatous infiltration of the mucosa and is rarely observed since it evolves without symptoms, in a latent fashion, and is only seen by accident. In the most cases the ulcerations vary in extent and number; in the most advanced cases they invade the vault of the pharynx and extend over the choanæ and even into the accessory sinuses and downward along the posterior pharynx.

The whole cavity is filled with a purulent mass mixed with blood. According to the duration and degree of the affection, the ulcers are superficial or deep; in the latter case, crater-like excavations are formed, reaching down to and into the bone, having their edges illy defined and presenting here and there some granulations. The favorite seat for an isolated ulceration is in the neighborhood of the pharyngeal bursal. The dirty grey deposit at the bottom of the ulcer adheres strongly and is removed with difficulty.

In examining with the probe the denuded bone is nearly always felt, but bone sequestra is much less here than in syphilis of the nose. Naso-pharyngeal syphilis, in spite of the general opinion to the contrary, presents sufficiently characterstic symptoms without any others. The patient complains of a sore throat which has lasted a long time and has resisted all the ordinary means of treatment, the pain is much aggravated in swallowing, and is in proportion to the extent of the ulcerations. Sometimes the pain is so great that rectal alimentation becomes necessary; accompanying pains in the head when persistent are pathognomonic. The occipital pain becomes so violent and long-continued that suicidal attempts have been made by many of the author's patients. The patient complains also of a great compression and fullness at the ears.

Another nearly pathognomonic symptom is the very abundant secretion composed of a purulent mass mixed with blood, of which the patient seeks to disembarrass himself by every possible means.

The author has examined one after the other of the sinuses thinking there might be a question of empyema, but without result.

It is necessary, says the author, in differential diagnosis to exclude diphtheria, tuberculosis and leprosy. In doubtful cases the secretions should be examined microscopically. The most difficult, from this point of view, are those cases with pronounced edema. The secretions are swallowed to provoke more or less gastric troubles; these, joined to the abstinence as a result of the dysphasia and insomnia result in a general feebleness, a veritable syphilitic cachexia so pronounced at first as to appear tuberculous.

When the ulcerations are confined to the naso-pharynx the odor is rarely fetid The diagnosis is usually easy, thanks to the presence of syphilitic manifestations elsewhere, but even in these cases a posterior rhinascopic examination must not be neglected, as ulcerations may persist here after all other symptoms have disappeared under the influence of specific treatment.

The pharyngeal bursa is the favorite seat for syphilitic ulcerations especially in scrofulous subjects, but in this situation deep ulcers are never found. In examining the naso-pharynx the posterior surface of the soft palate should never be forgotten, and above all should the vault of the pharynx be inspected since an ulceration in this situation might perforate the vertebral column. The treatment should be both general and local. The first consists of mercury and the iodides in alternate doses. The use of ether alone, in any form, will not give satisfaction, neither would the local treatment then be satisfactory. There have been observed some cases in which twenty, thirty or forty mercurial inunctions together with the iodides had been given without any good effect until systematic local treatment was begun.

The local treatment consists of daily cleansing of the whole cavity with sprays, douches and probes. After the cleansing the author throws through the cavity a solution composed of iodine, iodide of potash and glycerine, then an insufflation of powder composed of equal parts of calomel and orthoform.

The curette is recommended when granulations are too exhuberant, but too strong caustics are deplored.

The translator reported a unique case of syphilitic ulceration of the naso-pharynx before the Ninth International Medical Congress, at Washington, in 1887. The following is a short synopsis:

A Texas cowboy, age 25; weight 105; syphilitic skin manifestations; according to his history there had been no other lesion than the large, deep, crater-like ulcer in the middle upper post-pharyngeal wall. The ulcer cavity was filled with necrosed bone.

After a week of daily cleansing the spinal cord became visible to the size of the little finger nail. Pressure on the right side of the exposed cord with a cotton probe produced immediate paralysis of the entire left side of the body which passed off upon removal of the pressure and *vice versa* with pressure on the left side.

The cord felt to my finger very much as a moistened and slightly softened finger nail would feel. After the cicatrix had partially covered the exposed cord, pressure over its right side with the probe produced tetanic convulsions on the corresponding side of the body below the arms, and *vice versa* with pressure on the left side of the granulating wound. One may well imagine that these probe pressures were made with no small degree of solicitude, as there was no guiding precedent for probe pressing on the anterior column of a human being's spinal cord, although the results corresponded with the celebrated experiments of Brown Sequard on the lower animal.

None of the author's cases reached anything like the destructive stage of this case, no doubt because of his prompt treatment.

This case received no treatment until this advanced stage of destruction had been reached. At the end of six weeks the ulcer was healed under a rather heroic treatment, and at the end of three months the patient's weight was 135 pounds. Three hundred or 400 grains of iodide of potash was the daily dose for about ten days; once an ounce daily dose was reached and as much as half this amoun- daily before this period and for some time after it. He also had the mixed treatment. Other than antiseptic sprays and washes nothing was used locally but the cotton probe saturated in a strong solution of nitrate of silver for the purpose of excavating the sequestrum from the crater.

For a detailed report of this case see 4th vol. Transactions of the Ninth International Medical Congress. Since this rather unique case, my records show only eight others, similar to the author's fifteen cases, with the lesion confined to the naso-pharynx. Only one of these eight cases exhibited any bone sequestrum; all were completely cured with very large doses of iodide of potash together with the mixed treatment. But careful and systematic local treatment is quite as essential as the general treatment in obtaining good results in specific naso-pharyngeal ulcerations.

